# Identification and validation of three core genes in p53 signaling pathway in hepatitis B virus-related hepatocellular carcinoma

**DOI:** 10.1186/s12957-021-02174-w

**Published:** 2021-03-08

**Authors:** Mingxue Yu, Wenli Xu, Yusheng Jie, Jiahui Pang, Siqi Huang, Jing Cao, Jiao Gong, Xinhua Li, Yutian Chong

**Affiliations:** 1 Department of Infectious Diseases and Key Laboratory of Liver Disease of Guangdong Province, Third Affiliated Hospital of Sun Yat-sen University, Guangzhou, 510630 Guangdong Province China; 2 Department of Laboratory Medicine, Third Affiliated Hospital of Sun Yat-sen University, Guangzhou, 510630 Guangdong Province China

**Keywords:** Hepatocellular carcinoma (HCC), Hepatitis B virus (HBV), Biomarker, Bioinformatical analysis, p53 signaling pathway

## Abstract

**Background:**

Hepatocellular carcinoma (HCC) is a common cancer and the leading cause is persistent hepatitis B virus (HBV) infection. We aimed to identify some core genes and pathways for HBV-related HCC.

**Methods:**

Gene expression profiles of GSE62232, GSE121248, and GSE94660 were available from Gene Expression Omnibus (GEO). The GSE62232 and GSE121248 profiles were the analysis datasets and GSE94660 was the validation dataset. The GEO2R online tool and Venn diagram software were applied to analyze commonly differentially expressed genes between HBV-related HCC tissues and normal tissues. Then, functional enrichment analysis using Gene Ontology (GO) and the Kyoto Encyclopedia of Gene and Genome (KEGG) as well as the protein-protein interaction (PPI) network was conducted. The overall survival rates and the expression levels were detected by Kaplan-Meier plotter and Gene Expression Profiling Interactive Analysis (GEPIA). Next, gene set enrichment analysis (GSEA) was performed to verify the KEGG pathway analysis. Furthermore, quantitative reverse transcriptase polymerase chain reaction (qRT-PCR) was performed to validate the levels of these three core genes in tumor tissues and adjacent non-tumor liver tissues from 12 HBV related HCC patients, HBV-associated liver cancer cell lines and normal liver cell lines, and HepG2 with p53 knockdown or deletion, respectively.

**Results:**

Fifteen highly expressed genes associated with significantly worse prognoses were selected and CCNB1, CDK1, and RRM2 in the p53 signaling pathway were identified as core genes. GSEA results showed that samples highly expressing three core genes were all enriched in the p53 signaling pathway in a validation dataset (*P* < 0.0001). The expression of these three core genes in tumor tissue samples was higher than that in relevant adjacent non-tumor liver tissues (*P* < 0.0001). Furthermore, we also found that the above genes were highly expressed in liver cancer cell lines compared with normal liver cells. In addition, we found that the expression of these three core genes in p53 knockdown or knockout HCC cell lines was lower than that in negative control HCC cell lines (*P* < 0.05).

**Conclusions:**

CCNB1, CDK1, and RRM2 were enriched in the p53 signaling pathway and could be potential biomarkers and therapeutic targets for HBV-related HCC.

**Supplementary Information:**

The online version contains supplementary material available at 10.1186/s12957-021-02174-w.

## Introduction

Hepatocellular carcinoma (HCC) is a common cancer and the third leading cause of cancer death in the world [1, 2]. It has been reported that Chinese liver cancer patients account for more than 50% of all cases worldwide [3]. The major risk factors for HCC include chronic hepatitis B or C virus infection, alcoholic liver disease, and liver cirrhosis. The leading cause of HCC is persistent hepatitis B virus (HBV) infection, which happens in more than half of HCC case s[4, 5]. To date, many researchers have contributed to the identification of the underlying molecular mechanisms. However, the molecular pathogenesis and mechanisms of the hepatic carcinogenesis triggered by HBV is extremely complex, involving gene aberrations, mRNA expression, and the genome [6–8].

Various studies have shown that the occurrence and progression of liver cancer are closely related to the overexpression of oncogenes and the inactivation of tumor suppressor genes. Villanueva et al. [9] discovered many genes that are aberrantly methylated in HCC and observed that some signaling pathways are clearly deregulated by DNA methylation in HCC. Khemlina et al. also reported that genetic alterations in HCC include mutations in the TERT promoter [10]. Furthermore, Jiang et al. [11] found two genetic susceptibility loci for hepatitis B virus-related HCC. The Janus Kinase (JAK)/STAT pathway and Wnt/β-catenin pathway may play roles in HCC acting as two major oncogenic drivers, which might serve as potential treatment targets [12, 13].

With the rapid development of high-throughput DNA microarray technology, gene expression microarray analysis has emerged as a promising and efficient tool to help with an understanding of the precise underlying mechanisms in cancer. Biomarkers have been identified and confirmed to potentially improve the diagnosis and therapy of HCC [14, 15]. Recently, some gene expression profiling microarrays have been used to identify various differentially expressed genes (DEGs) in HCC [16–19]. However, few reliable biomarkers have been identified in hepatitis B virus (HBV)-related hepatocellular carcinoma. It is critical to explore more effective biomarkers for the development and recurrence of HBV related HCC. We took the initiative to combine integrated bioinformatics methods and validation experiments to investigate some core genes and underlying molecular mechanisms.

In this study, the common DEGs in datasets GSE62232 and GSE121248 were used in the analysis. Core genes were identified based on bioinformatic analysis. Then, gene set enrichment analysis (GSEA) was performed in the validation dataset GSE94660. Subsequently, we validated the expression of core genes in liver cancer tissue samples and liver cancer cell lines using quantitative reverse transcriptase polymerase chain reaction (qRT-PCR).

## Materials and methods

### Microarray data

GSE62232, GSE121248, and GSE94660 gene expression profiles were downloaded from the Gene Expression Omnibus [20] (GEO, https://www.ncbi.nlm.nih.gov/geo), which included 10 HBV-related HCC tissues and 10 normal tissues, 70 HBV-related HCC tissues and 37 normal issues, and 21 pairs of tumor and non-neoplastic liver tissues of HBV-HCC patients, respectively. The GSE62232 and GSE121248 profiles were the analysis datasets and GSE94660 was the validation dataset.

### Data processing of differentially expressed genes (DEGs)

The identification of DEGs between HCC specimens and normal specimens was performed in GEO2R (https://www.ncbi.nlm.nih.gov/geo/geo2r/) [20]. The DEGs were screened according to adjusted *P* values < 0.05 and |logFC| > 2. Then, the common DEGS expressed jointly in the two data files were detected with Venn software. The DEGs with log FC < 0 were considered downregulated genes, while the DEGs with log FC > 0 were considered upregulated genes.

### Gene ontology (GO) and Kyoto Encyclopedia of Genes and Genomes (KEGG) pathway enrichment analysis

The Database for Annotation, Visualization, and Integrated Discovery (DAVID Version 6.8, https://david.ncifcrf.gov/) [21] and KOBAS 3.0 (http://kobas.cbi.pku.ed-u.cndate) [22], which are online bioinformatic tools with a threshold of FDR < 0.05 and *P* < 0.05, were used to analyze the DEGs of GO [23], enrichment of BP (biological process), MF (molecular function), and CC (cellular component) and KEGG pathways [24]. Data processing and the creation of graphics were performed with “ggplot2” R package in R software (Version 3.4.0, https://www.r-project.org/).

### Construction of protein-protein interaction (PPI) network

STRING online database (http://string-db.org )[25] was applied to detect core candidate genes. Cytoscape software [26] (Version 3.4.0, http://www.cytoscape.org/) was used to construct a PPI relationship network. In addition, the plugin MCODE in Cytoscape was utilized to identify crucial genes of highly intraconnected nodes (degree cutoff ≥ 2, node score cutoff ≥ 0.2, Kcore ≥ 2, and max depth = 100).

### Survival analysis and expression level of core genes

Kaplan Meier-plotter [27] (http://www.kmplot.com) was applied to perform a survival analysis of the core genes. The hazard ratio (HR) with 95% confidence intervals and logrank *p* value were calculated and displayed on the plot. We also used the GEPIA website [28] (Gene Expression Profiling Interactive Analysis, http://gepia.cancer-pku.cn/index.html) to analyze the data of RNA sequencing expression on the basis of samples from the GTEx and TCGA (https://cancergenom e.nih.gov/).

### Gene set enrichment analysis (GSEA)

Gene Set Enrichment Analysis (GSEA) is a computational method that assesses whether an a priori defined set of genes shows statistically significant, concordant differences between two biological states. We used the version of the Java based software (GSEA-P 2.0) [29] (http://software.broadinstitute.org/gsea/index.jsp) to detect functional analysis between the two groups derived from DEGs. In this study, briefly, samples of HBV-HCC in validation set GSE94660 were divided into two groups according to the expression level of the three core genes (*CCNB1*, *CDK1*, and *RRM2*) [30]. For groups, HBV-HCC samples were divided into subgroups with high or low expression of three core genes based on Reads Per Kilo bases per Million reads (RPKM) data. Gene symbol and RPKM data were sorted in descending order to create the ranking list. The top 20 samples were extracted as the high expression while the others as low expression [31]. Then, GSEA was applied to detect whether the p53 signaling pathway was enriched in the highly expressed core gene of HBV-HCC samples. Terms with *P* < 0.01 were identified.

### Cell culture and transfection of RNA

All three HBV-related liver cell lines (HepG2.215, HepG-AD-38, and HepG-DE-19) were obtained from the cell bank of the Chinese Academy of Sciences (Shanghai, China). HepG2 and HepG2-KO-p53 cell lines were gifts from Professor Lianghu Qu (State key Laboratory of Biocontrol, Sun Yat-sen University, Guangzhou, China). Cells were cultured in MEM medium containing 10% fetal bovine serum plus antibiotics (GIBICO). Cells were maintained in a 5% CO_2_ atmosphere at 37 °C. HepG2 cells were transfected with p53 siRNAs at a final concentration of 50 nM using Lipofectamine 2000 (Invitrogen) according to the manufacturer’s instructions. The p53 siRNAs used in this study were purchased from GenPharma, and the sequence of the p53-siRNA was AGACCTATGGAAACTACTT.

### The collection of human liver tissue samples

This study was approved by the Ethics Committee of the Third Affiliated Hospital of Sun Yat-sen University ([2015]2-206 No.1). Paired HBV-HCC tissues (T) and their corresponding adjacent non-tumor liver tissues (NT) were obtained from HBV-related HCC patients at the Third Affiliated Hospital of Sun Yat-Sen University. The main characteristics of the patients’ clinical data are shown in Table 1, which included their age, sex, pathological type, size of tumor, BCLC stage, HBsAg, HBV-DNA, alpha fetoprotein (AFP), alanine aminotransferase (ALT), and aspartate aminotransferase (AST). Informed consent was obtained from all participants included in the study, and experimental procedures were performed according to the guidelines of the non-profit, state-controlled HTCR (Human Tissue and Cell Research) foundation.

**Table 1:** Clinical characteristic of the hepatocellular carcinoma patients

Patient ID	Age	Sex	Pathological type	Size of tumor	BCLC stage	HBsAg (IU/mL)	HBV-DNA (IU/mL)	AFP (ng/mL)	ALT(U/L)	AST(U/L)
1	58	M	Moderately differentiated	32 × 22 mm	A	> 180	8.69E+3	1073	40.7	33.3
2	47	M	Poorly differentiated	28 × 25 mm	A	29.86	1.06E+2	1.43	248.2	183.2
3	64	M	Moderately differentiated	77 × 71 mm	B	> 180	1.08E+5	110	25.8	55.1
4	60	M	Moderately to poorly differentiated	106 × 73 mm	C	159.58	3.24E+2	16.09	33.6	46
5	61	M	Moderately differentiated	16 × 17 mm	O	> 250	5.89E+2	766.5	16	12.9
6	46	M	Moderately to poorly differentiated	27 × 28 mm	A	> 180	2.60E+5	4371	37.2	25.7
7	38	M	Moderately differentiated	25 × 24 mm	A	251.9	< 100	0.746	35	43
8	76	M	S8:Well-differentiated S7:Moderately to poorly differentiated	15 × 13 mm	O	1.17	< 100	unknown	28	28
9	57	M	Moderately differentiated	27 × 19 mm	A	> 250	5.05E+03	3.129	20	16
10	64	M	Well-differentiated	24 × 20 mm	A	2165	< 100	5.06	24	22
11	53	M	Moderately differentiated	62 × 63 mm	B	1070	4.21E+05	3.516	32	53
12	45	M	Moderately differentiated	81 × 51 mm	B	> 180	1.21E+2	855.5	23.5	19.2

### RNA extraction and quantitative reverse transcriptase polymerase chain reaction (qRT-PCR)

The total RNA was isolated from the cell lines and the HBV related liver tissues using Trizol reagent (Invitrogen). cDNA was synthesized using a PrimeScript™ RT Reagent Kit with a gDNA Eraser Kit (Takara). Human normal liver tissue RNA was purchased from Clontech. PCR amplifications were constructed with the SYBR® Premix Ex Taq™ II (Takara) and normalized GAPDH for comparison. The ΔΔCt method for the relative quantitation (RQ) of gene expression was used to determine *CCNB1*, *CDK1*, *RRM2*, *p53*, *p21*, and *PUMA* expression levels. The corresponding primers used in this study are listed in Table 2.

**Table 2:** Sequences of primers and oligos

Gene name	Forward	Reverse
GAPDH	CCATGGGGAAGGTGAAGGTC	GAAGGGGTCATTGATGGCAAC
CCNB1	GTGGATGCAGAAGATGGAGC	CCGACCCAGTAGGTATTTTGG
CDK1	AGGAAGGGGTTCCTAGTACTGC	TGGAATCCTGCATAAGCACA
RRM2	TCTATGGCTTCCAAATTGCC	GACACAAGGCATCGTTTCAA
p53	TAACAGTTCCTGCATGGGCGGC	AGGACAGGCACAAACACGCACC
p21	TGGAGACTCTCAGGGTCGAA	GGATTAGGGCTTCCTCTTGG

### Statistical analysis

Statistical analysis was conducted using GraphPad Prism 8 and *P* < 0.05 was considered statistically significant. All results were expressed as the mean ± standard error of the mean (SEM). A paired Student’s *t* test was applied for comparisons between the two experimental groups. All experiments were performed at least three times.

## Results

### Identification of DEGs in hepatitis B virus-related hepatocellular carcinoma

**Fig. 1:**
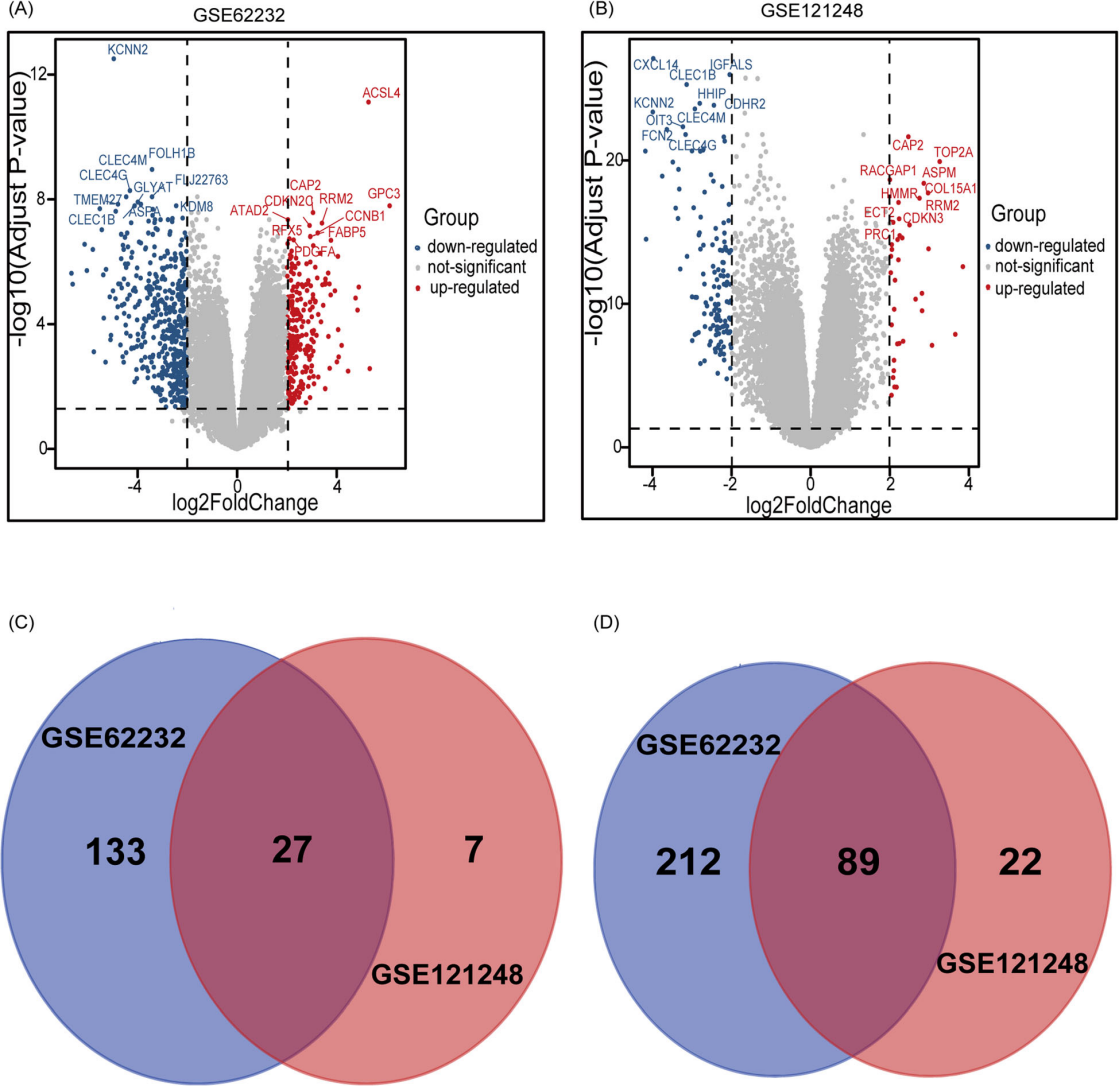
Volcano plots and Venn diagrams of differentially expressed genes. **a** Volcano plots of GSE62232. **b** Volcano plots of GSE121248. Each colored dot represents an up or downregulated gene, where blue indicates genes with low levels of expression, red indicates genes with high levels of expression, and gray indicates genes with no differential expression based on the criteria of *P*< 0.05 and |log FC| > 2. **c** 27 DEGs were upregulated in the two datasets (logFC> 0). **d** 89 DEGs were downregulated in two datasets (logFC< 0). Different color meant different datasets

A total of 606 DEGs were extracted from GSE62232 and GSE121248 between HBV-related HCC tissues and normal tissues with adjusted *P* < 0.05 and |logFC| > 2. The volcano plots of DEGs in the two datasets are shown in Fig. 1. The top 10 genes that were up- or downregulated were marked with text in the volcano plots. The Venn diagram showed that a total of 116 common DEGs were detected, including 89 downregulated genes and 27 upregulated genes in the datasets (Table 3, Fig. 1c, d).

**Table 3:** Commonly DEGs in GSE62232 and GSE121248

DEGs	Gene name
Upregulated	CDK1 SPINK1 CAP2 DTL RACGAP1 CTHRC1 RRM2 IGF2BP3 CCNB TOP2A ASPM HMMR CDKN3 AKR1B10 PBK GPC3 ROBO1 SPP1 ZIC2 NEK2 ANLN ACSL4 CRNDE BUB1B COL15A1 ECT2 PRC1
Downregulated	CYP4A22///CYP4A11 CYP26A1 BBOX1 CYP2A6 CNTN3 TENM1 LINC01093 CXCL14 SLC22A1 IGF1 SULT1E1 CYP39A1 HAO2 FAM134B MT1F SLC25A47 MFSD2A ZG16 FLJ22763 HHIP KCNN2 ZGPAT///LIME1 SLCO1B3 CYP1A2 CNDP1 BCO2 ACSM3 FCN3 GBA3 TTC36 CLEC4G C3P1 CDH19 CYP2B6 GYS2 FOLH1B KMO LPA CD5L GHR CLEC1B MIR675///H19 CXCL2 LIFR FAM65C CLRN3 CYP2C9 CYP2A7 LCAT CLEC4M VNN1 ESR1 LOC101928916///NNMT PLAC8 ALDOB HAMP DNASE1L3 DCN NAT2 BCHE IL1RAP AKR1D1 CXCL12 TMEM27 CRHBP TACSTD2 WDR72 THRSP IDO2 HGFAC IGFALS ADGRG7 ZGPAT FREM2 ADH4 GPM6A OIT3 HGF MT1M GLYAT CYP2B7P///CYP2B6 GLS2 SRD5A2 ADRA1A APOF C9 SRPX FCN2 LINC00844

### GO and KEGG pathway enrichment analysis

**Fig. 2:**
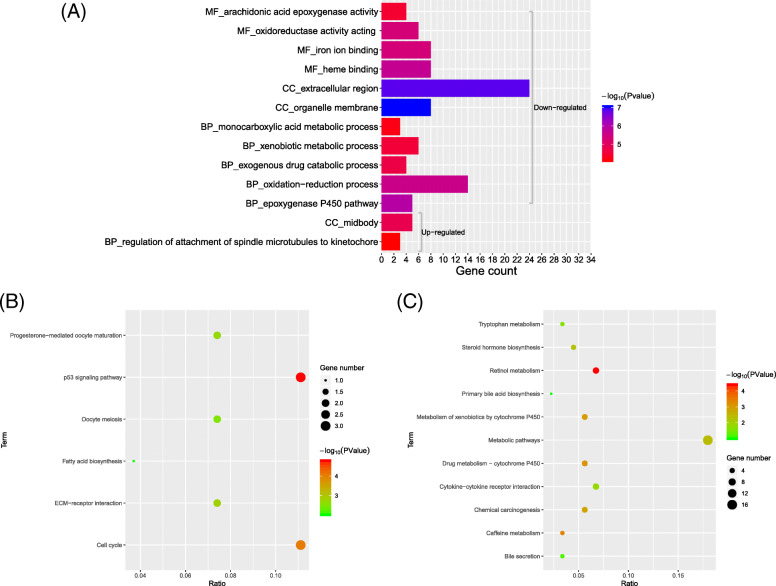
GO and KEGG pathway analysis of differentially expressed genes. **a** GO analysis of DEGs. GO analysis included enrichment of BP (biological process), MF (molecular function), and CC (cellular component). The gray bracket below indicates analysis of upregulated DEGs, while bracket on the top indicates analysis of downregulated genes. **b** KEGG pathway analysis of upregulated DEGs. **c** KEGG pathway analysis of downregulated DEGs

The candidate DEGs were classified into three functional groups, consisting of the biological process (BP) group, cellular component (CC) group, and molecular function (MF) group with FDR < 0.05 and *P* < 0.05. The results demonstrated that for the upregulated DEGs, the BPs included “regulation of attachment of spindle microtubules to kinetochore.” For downregulated DEGs, the top 5 BPs were “epoxygenase P450 pathway,” “oxidation-reduction process,” “exogenous drug catabolic process,” “xenobiotic metabolic process,” and “monocarboxylic acid metabolic process.” In the CC analysis, it was revealed that upregulated DEGs were predominantly involved in “midbody,” while downregulated DEGs were involved in “organelle membrane” and “extracellular region.” Moreover, in the MF analysis, downregulated DEGs were associated with “heme binding,” “iron ion binding,” “oxidoreductase activity,” and “arachidonic acid epoxygenase activity” while upregulated DEGs were in no significant signaling pathways (Fig. 2a and Supplementary Table 1)

KEGG analysis results indicated that the top five KEGG pathways for upregulated DEGs were “p53 signaling pathway,” “Cell cycle,” “ECM-receptor interaction,” “Progesterone-mediated oocyte maturation,” and “Oocyte meiosis,” while for downregulated DEGs, they were “Retinol metabolism,” “Caffeine metabolism,” “Drug metabolism-cytochrome P450,” “Metabolism of xenobiotics by cytochrome P450,” and “chemical carcinogenesis” (*P* < 0.05, Fig. 2b, c).

### Protein-protein interaction (PPI) network of DEGs

**Fig. 3:**
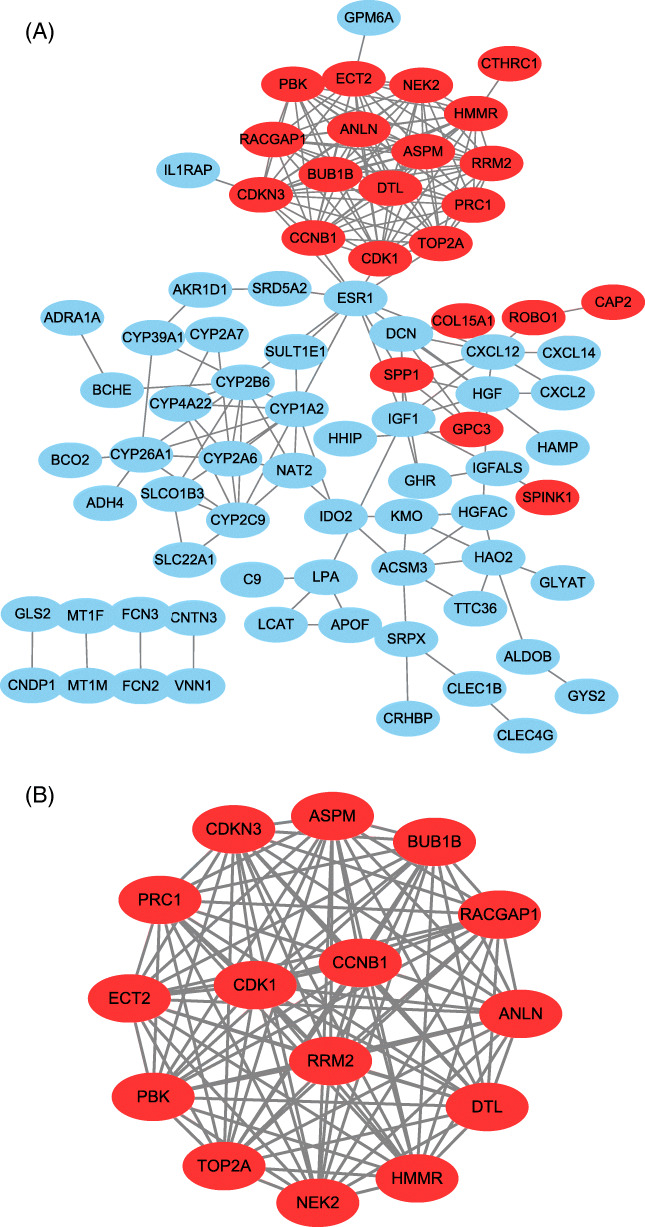
Protein-protein interaction (PPI) network of DEGs. Common DEGs PPI network constructed by STRING online database and Module analysis. **a** There were a total of 78 DEGs in the DEGs PPI network complex. The nodes meant proteins, the edges meant the interaction of proteins, blue circles meant downregulated DEGs, and red circles meant upregulated DEGs. **b** Module analysis via Cytoscape software (degree cutoff = 2, node score cutoff = 0.2, k-core = 2, and max. Depth = 100)

Seventy-eight of the 116 DEGs were filtered into the construction of a PPI network, including 56 downregulated and 22 upregulated genes (Fig. 3a). The network contained 78 nodes and 209 interactions. To detect the most significant module in the PPI network, the results of the Cytotype MCODE analysis demonstrated that 15 central nodes that had the highest degree genes were identified among the 78 nodes. These 15 central nodes were as follows: *CDK1*, *PRC1*, *NEK2*, *DTL*, *ANLN*, *PBK*, *RACGAP1*, *CDKN3*, *ECT2*, *HMMR*, *CCNB1*, *RRM2*, *BUB1B*, *TOP2A*, and *ASPM*, which were all upregulated DEGs (Fig. 3b).

### Survival analysis and expression level of selected genes

**Fig. 4:**
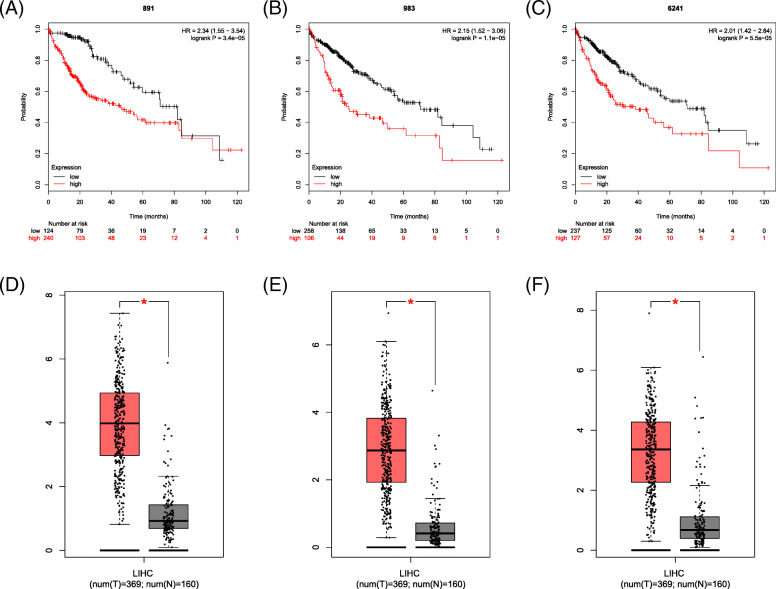
The prognostic information and expression level of three core genes. **a**, **d** CDK1. **b**, **e** CCNB1. **c**, **f** RRM2

The prognostic information of the fifteen selected genes was available in the Kaplan Meier Plotter database. As shown in Fig. 4 and Supplementary Figure 1, the fifteen selected genes were all associated with a poor prognosis for HCC (*P* < 0.05). To further identify the genes expression level, GEPIA was used to validate the expression levels of the genes. The results showed that all 15 selected genes had high expression levels in HCC samples (*P* < 0.05; Fig. 4 and Supplementary Figure 2).

### KEGG pathway enrichment re-analysis of 15 selected genes

**Fig. 5:**
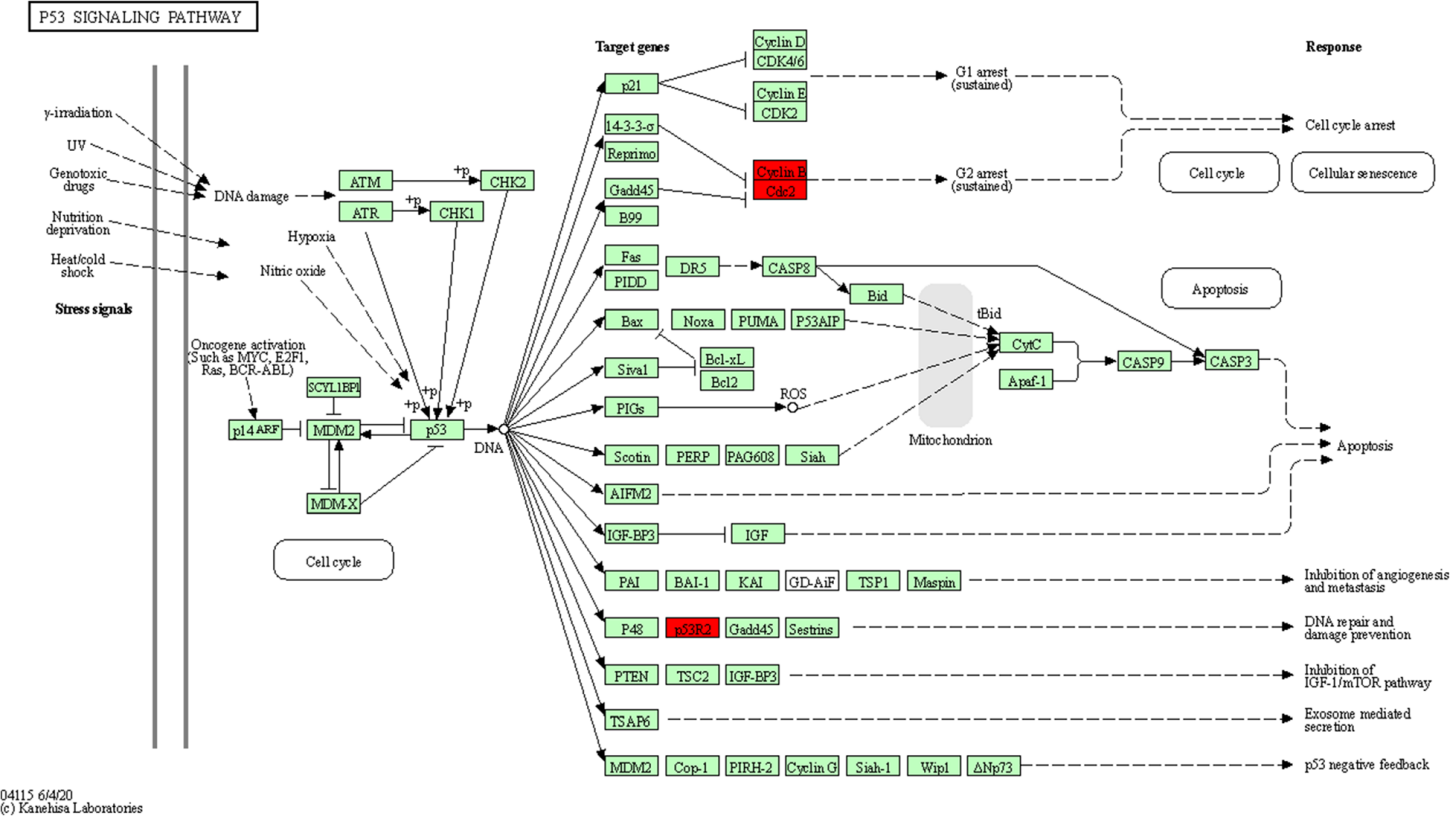
Re-analysis of 15 selected genes via KEGG pathway enrichment analysis. Fifteen high expressed genes in liver cancer with poor prognosis were re-analyzed by KEGG pathway enrichment. Three genes (*CCNB1*, *CDK1*, and *RRM2*) were significantly enriched in the p53 signaling pathway (*P =* 2.55E-06). Cyclin B means CCNB1. CDC2 means CDK1. P53R2 means RRM2

Furthermore, we re-analyzed these 15 genes in KEGG pathway enrichment via KOBAS 3.0. The results showed that the p53 signaling pathway was the most significant pathway (*P* = 2.55E−06, Fig. 5) and three core genes (CCNB1, CDK1, and RRM2) were significantly enriched in this pathway.

### Gene set enrichment analysis (GSEA) in GSE94660

**Fig. 6:**
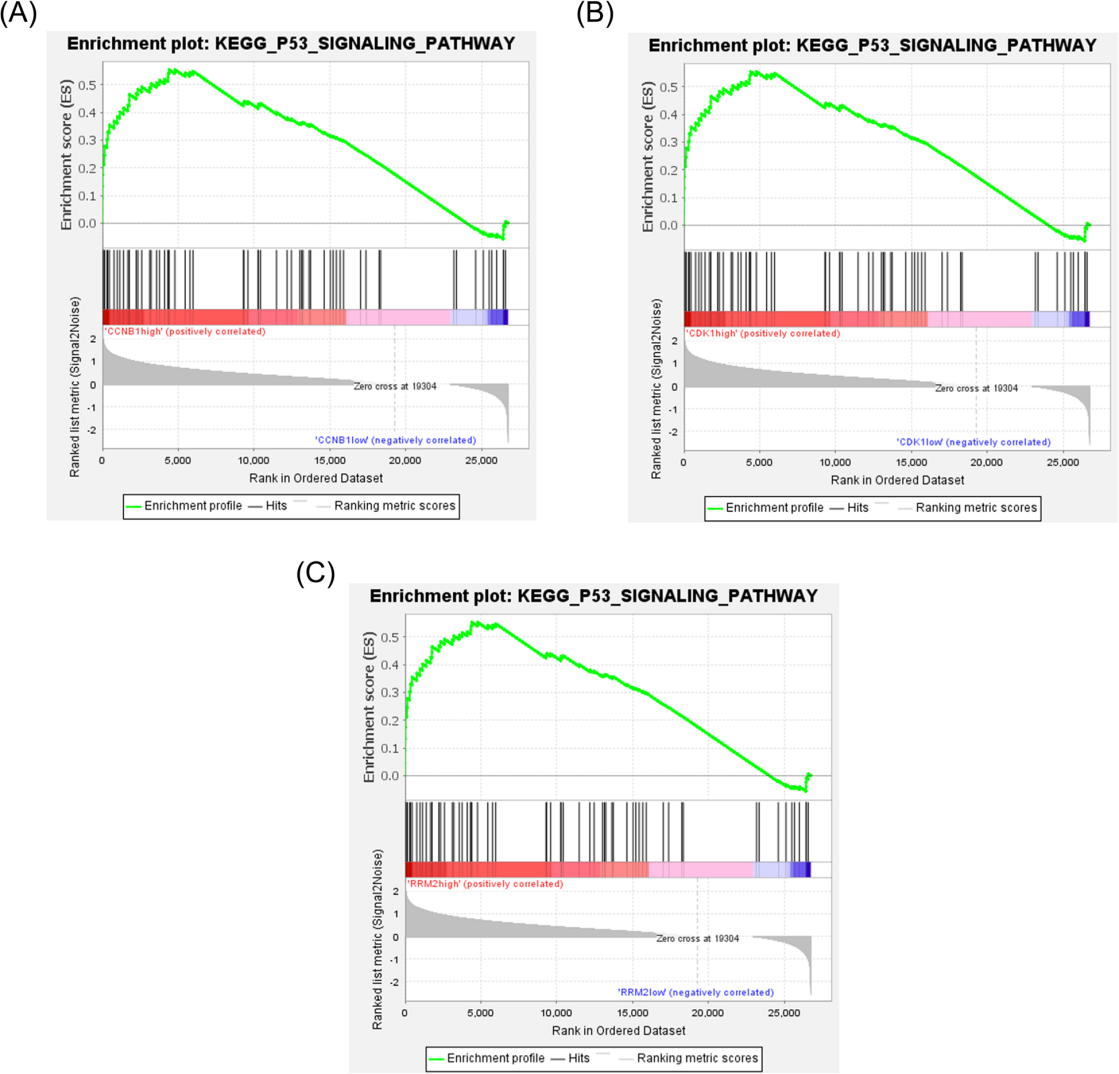
Gene set enrichment analysis (GSEA) in GSE94660. Highly expressed HBV-HCC samples of three core genes were all enriched in p53 signaling pathway (*P* < 0.0001). **a** CCNB1. **b** CDK1. **c** RRM2

To verify whether the p53 signaling pathway was enriched in the highly expressed core gene samples, GSEA was used in the validation dataset GSE94660. We divided the samples from HBV-HCC into two groups according to the expression levels of the three core genes. The results showed that samples highly expressing *CCNB1*, *CDK1*, and *RRM2* respectively were all enriched in the p53 signaling pathway in a validation dataset (*P* < 0.001; Fig. 6).

### Validation gene expression in liver cancer tissues and cell lines

**Fig. 7:**
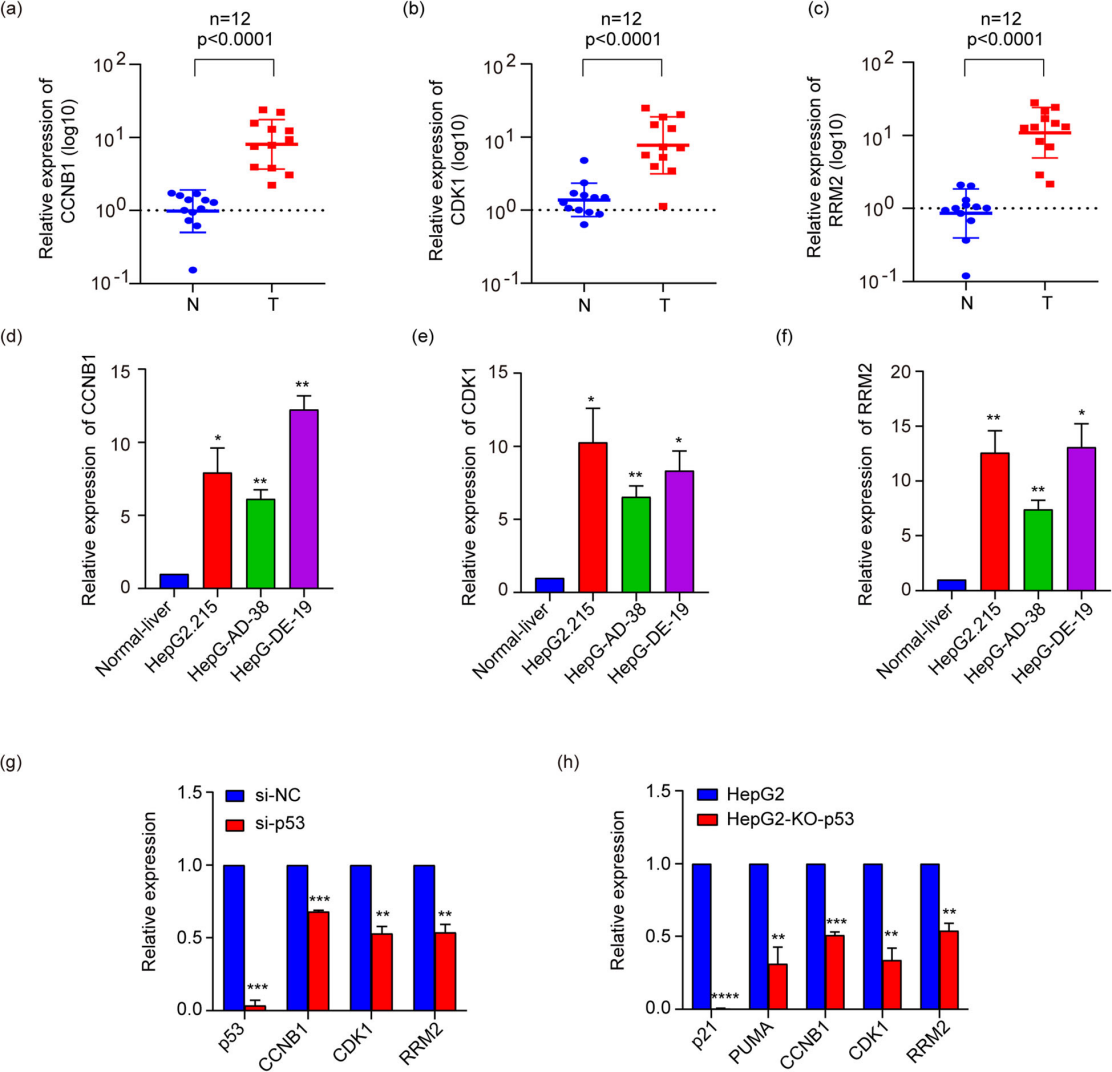
qRT-PCR results in liver tissues and liver cancer cell lines. The expression level of CCNB1, CDK1, and RRM2 in liver cancer tissues (**a**–**c**). All of three genes were significantly upregulated in tumor tissues compared with adjacent normal tissues (*P* < 0.0001). The expression level of CCNB1, CDK1, and RRM2 in HBV-related liver cancer cell lines (**d**–**f**). **g** Cells were transfected with negative control siRNA (si-NC) or siRNAs against p53(si-p53). **h** Knockdown of p53 HepG2 cells and HepG2 cells. Results indicated significant difference between groups (*P < 0.05, **P < 0.01, ***P < 0 .001, ****P < 0.0001).

In order to verify the expression of the core genes, we used qPCR to detect their expression in liver cancer tissues and cells. The qRT-PCR results showed that *CCNB1*, *CDK1*, and *RRM2* were significantly upregulated in liver cancer tumor samples (*P* < 0.0001; Fig. 7a–c) and cell lines (*P* < 0.05; Fig. 7d–f) compared with the relevant adjacent non-tumor liver tissues and normal liver cell LO2. Furthermore, we found that knockdown or knockout p53 could significantly inhibit the expression of *p53*, classical p53 target genes (*p21* and *PUMA*) [32, 33], and these three genes (Fig.7g, h). These results indicated that *CCNB1*, *CDK1* and *RRM2* were highly expressed in liver cancer tissues and liver cancer cell lines and enriched in the p53signaling pathway.

## Discussion

In the present study, we explored a total of 116 common DEGs comprising 27 upregulated and 89 downregulated DEGs. Then, DAVID together with KOBAS was used to analyze GO and KEGG pathways, and PPIs of these DEGs were visualized with Cytoscape. The PPI network complex of 78 nodes and 209 edges was constructed. According to the results of Cytotype MCODE, 15 upregulated genes were identified from the PPI network complex. We found that CDK1, PRC1, NEK2, DTL, ANLN, PBK, RACGAP1, CDKN3, ECT2, HMMR, CCNB1, RRM2, BUB1B, TOP2A, and ASPM had a significantly worse survival rate and high expression in the HCC samples. Furthermore, KEGG pathway enrichment re-analysis results of the 15 selected genes indicated that CCNB1, CDK1, and RRM2 were significantly enriched in the p53 signaling pathway. In GSEA analysis, highly expressed HBV-HCC samples of three core genes were enriched in the p53 signaling pathway in the validation dataset. Our bioinformatics analysis results predicted that CCNB1, CDK1, and RRM2 may be closely related to the development of HBV-related HCC. The verification results in liver cancer tissues and cells showed that expression of the core genes was higher than in the normal tissues and cells, while transfection of si-p53 and knockdown of p53 led to lower expression, indicating that three core genes in the p53 signaling pathway may play a significant role in the occurrence and development of HBV-related HCC.

*CCNB1*, G2/Mitotic-specific cyclin B1, was shown to play an important role in the occurrence and development of tumors. Mussnich reported that downregulation of CCNB1 could reduce cell proliferation [34]. Zhao [35] reported that upregulation of CCNB1 played a part in the pathology of pituitary adenomas in the cell cycle. CDK1, cyclin-dependent kinases A, was suggested to play a role in the development of HCC. Some results strongly suggested that CDK1 acts as a tumor-specific mediator, affecting apoptin-induced cytotoxicity in HCC cells. CDK1 could be an important factor in cell division [36], and several CDK1 substrates, such as histone H1 and PI3K/AKT, play crucial roles in cell cycle modulation [37, 38].

*RRM2*, ribonucleotide reductase regulatory subunit M2, is one of the protein genes encoding ribonucleoreductase (RR) [39]. *RRM2* can be used as a prognostic biomarker for a variety of cancer types, such as colon cancer and breast cancer [40–42], and overexpression of *RRM2* also stimulates the migration, invasion, and proliferation of many other solid tumor cells. A previous study showed that *RRM2* is directly regulated by p53 to supply nucleotides to repair damaged DNA [43]. A study also showed that p53 R2-dependent DNA synthesis plays a pivotal role in cell survival by repairing damaged DNA in the nucleus and that dysfunction of this pathway might result in activation of p53-dependent apoptosis to eliminate dangerous cells [44]. Small-interfering RNA-mediated knockdown of RRM2 can depress HCC cell proliferation [45]. Furthermore, Lee [46] indicated that high expression of RRM2 could be a useful marker to predict early recurrence of HCC following curative hepatectomy.

Moreover, the p53 signaling pathway was a potent barrier for tumor progression and it plays important roles in hepatocarcinogenesis [47–50]. Tu [51] found that the activation of F-box and WD repeat domain-containing 7 (Fbxw7) via adenoviral delivery of p53 caused increased proteasomal degradation of cyclin E and c-Myc, thus recombinant human adenovirus-p53 injection could be a possible therapeutic agent for HCC.

A large number of studies have shown that these three genes and p53 signaling pathway were related to the progression of various cancers. However, few studies have proved the role of these three genes in HBV related hepatocellular carcinoma. Therefore, our study can provide useful information and direction for the study of liver cancer and needs further research and experimental verification.

## Conclusions

Considering the above, using a series of bioinformatics analysis and validation experiments, our results suggested that *CCNB1*, *CDK1*, and *RRM2* may be key genes and the p53 signaling pathway may play a vital role in the development of HBV-related HCC.

## Supplementary Information

**Additional file 1: Supplementary Table 1.** Gene ontology analysis of differentially expressed genes in HBV-HCC The original results of GO analysis DEGs, enrichment of BP (biological process), MF (molecular function), and CC (cellular component) by using DAVID with FDR <0.05 and *P* < 0.05.

**Additional file 2: Supplementary figure 1.** The prognostic information of other 12 genes (a) ANLN (b)ASPM (c)BUB1B (d)CDKN3 (e)DTL (f)ECT2 (g)HMMR (h)NEK2 (i)PBK (j)PRC1 (k)RACGAP1 (l)TOP2A.

**Additional file 3: Supplement figure 2.** The expression level of other 12 genes (a)ANLN (b)ASPM (c)BUB1B (d)CDKN3 (e)DTL (f)ECT2 (g)HMMR (h)NEK2 (i)PBK (j)PRC1 (k)RACGAP1 (l)TOP2A.

## Data Availability

All data generated or analyzed during this study are included in this published article.
