# Supplementary material for: Identification and validation of three core genes in p53 signaling pathway in hepatitis B virus-related hepatocellular carcinoma
Source: World J Surg Oncol. 2021 Mar 8;19:66. doi: 10.1186/s12957-021-02174-w (PMC7938465; doi:10.1186/s12957-021-02174-w)
Supplement: Supplementary file 1 — Additional file 1: Supplementary Table 1. Gene ontology analysis of differentially expressed genes in HBV-HCC The original results of GO analysis DEGs, enrichment of BP (biological process), MF (molecular function), and CC (cellular component) by using DAVID with FDR <0.05 and P < 0.05. [file 12957_2021_2174_MOESM1_ESM.docx]

**Supplementary Table 1 Gene ontology analysis of differentially expressed genes in HBV-HCC**

| Expression | Category | Term | | Count | | | P | % | FDR |
| --- | --- | --- | --- | --- | --- | --- | --- | --- | --- |
| Up-regulated | GOTERM_BP_DIRECT | GO:0051988~regulation of spindle microtubules to kinetochore | | 3 | | 7.94E-05 | | 0.067689531 | 0.094128461 |
|  | GOTERM_CC_DIRECT | GO:003049 attachment 6~midbody | | 5 | 1.48E-05 | | | 0.112815884 | 0.013800407 |
| Down-regulated | GOTERM_BP_DIRECT | GO:0019373~epoxygenase P450 pathway | 5 | | 1.19E-06 | | | 0.050145422 | 0.001753641 |
|  | GOTERM_BP_DIRECT | GO:0055114~oxidation-reduction process | 14 | | 2.64E-06 | | | 0.140407181 | 0.003882104 |
|  | GOTERM_BP_DIRECT | GO:0042738~exogenous drug catabolic process | 4 | | 1.98E-05 | | | 0.040116337 | 0.029148787 |
|  | GOTERM_BP_DIRECT | GO:0006805~xenobiotic metabolic process | 6 | | 2.89E-05 | | | 0.060174506 | 0.04254496 |
|  | GOTERM_BP_DIRECT | GO:0032787~monocarboxylic acid metabolic process | 3 | | 6.21E-05 | | | 0.030087253 | 0.091373569 |
|  | GOTERM_CC_DIRECT | GO:0031090~organelle membrane | 8 | | 8.88E-08 | | | 0.080232675 | 9.76E-05 |
|  | GOTERM_CC_DIRECT | GO:0005576~extracellular region | 24 | | 1.45E-07 | | | 0.240698024 | 1.59E-04 |
|  | GOTERM_MF_DIRECT | GO:0020037~heme binding | 8 | | 2.07E-06 | | | 0.080232675 | 0.002625359 |
|  | GOTERM_MF_DIRECT | GO:0005506~iron ion binding | 8 | | 4.32E-06 | | | 0.080232675 | 0.005474573 |
|  | GOTERM_MF_DIRECT | GO:0016705~oxidoreductase activity acting | 6 | | 4.63E-06 | | | 0.060174506 | 0.005857379 |
|  | GOTERM_MF_DIRECT | GO:0008392~arachidonic acid epoxygenase activity | 4 | | 3.40E-05 | | | 0.040116337 | 0.043056745 |
