# Supplementary figures and images for: Identification and validation of three core genes in p53 signaling pathway in hepatitis B virus-related hepatocellular carcinoma
Source: World J Surg Oncol. 2021 Mar 8;19:66. doi: 10.1186/s12957-021-02174-w (PMC7938465; doi:10.1186/s12957-021-02174-w)

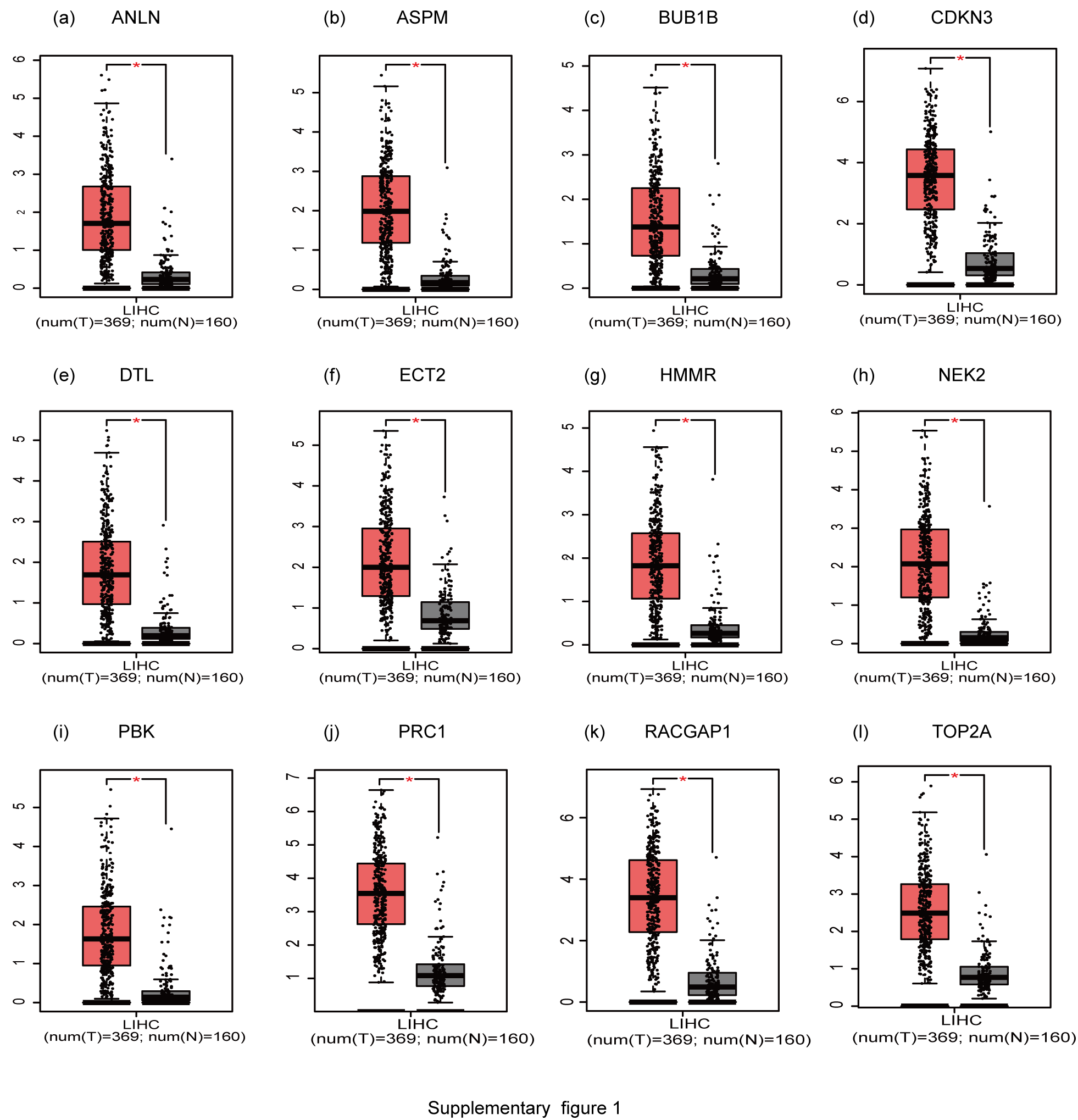

Supplement: Supplementary file 2 — Additional file 2: Supplementary figure 1. The prognostic information of other 12 genes (a) ANLN (b)ASPM (c)BUB1B (d)CDKN3 (e)DTL (f)ECT2 (g)HMMR (h)NEK2 (i)PBK (j)PRC1 (k)RACGAP1 (l)TOP2A. [file 12957_2021_2174_MOESM2_ESM.tif]

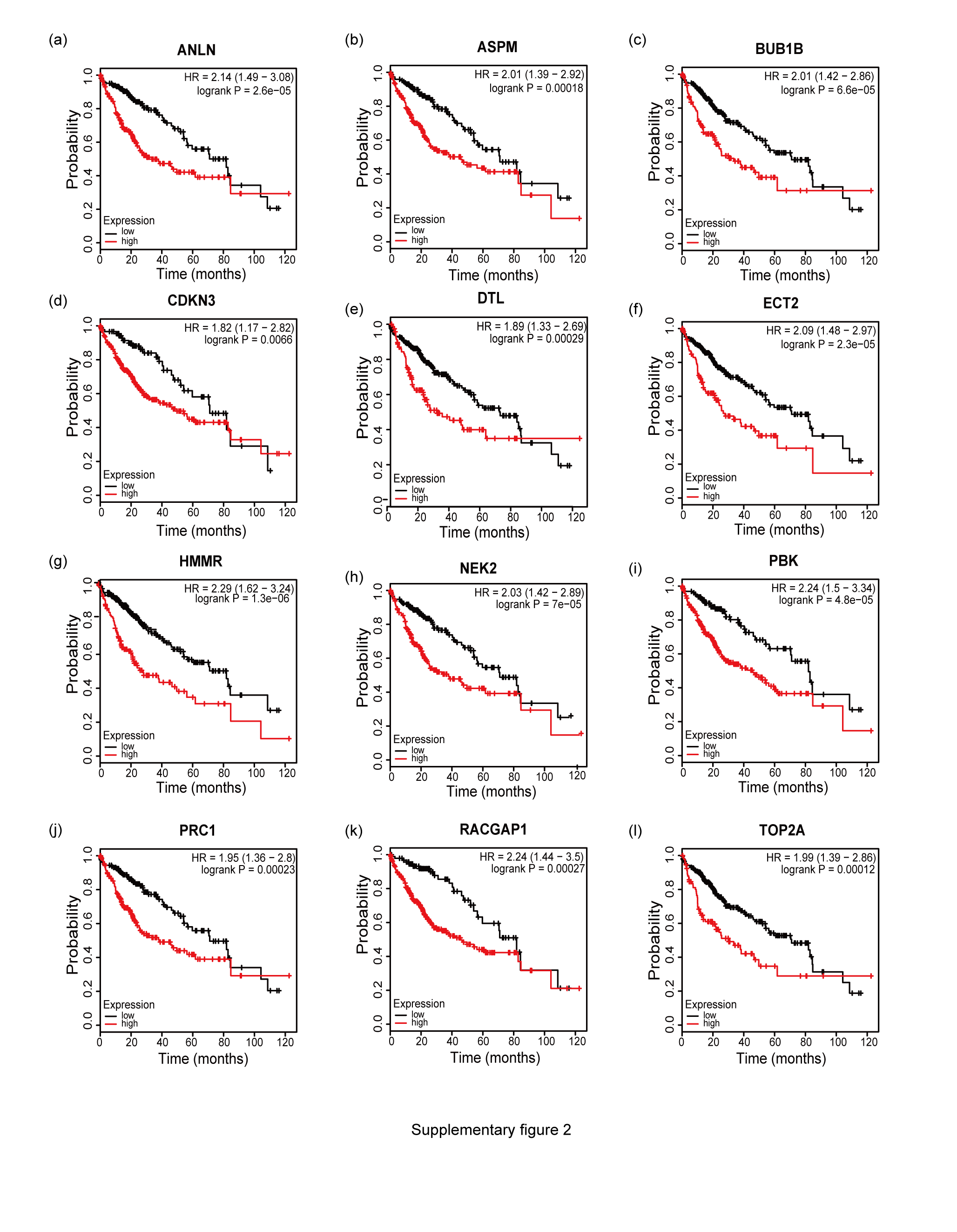

Supplement: Supplementary file 3 — Additional file 3: Supplement figure 2. The expression level of other 12 genes (a)ANLN (b)ASPM (c)BUB1B (d)CDKN3 (e)DTL (f)ECT2 (g)HMMR (h)NEK2 (i)PBK (j)PRC1 (k)RACGAP1 (l)TOP2A. [file 12957_2021_2174_MOESM3_ESM.tif]
